# Prevalence and Trends of Slow Gait Speed in the United States

**DOI:** 10.3390/geriatrics8050095

**Published:** 2023-09-25

**Authors:** Emily Stover, Sarah Andrew, Joshua Batesole, Maren Berntson, Chloe Carling, Samantha FitzSimmons, Tyler Hoang, Joseph Nauer, Ryan McGrath

**Affiliations:** 1 Healthy Aging North Dakota (HAND), North Dakota State University, Fargo, ND 58102, USA; 2 Department of Health, Nutrition, and Exercise Sciences, North Dakota State University, Fargo, ND 58108, USA

**Keywords:** geriatrics, physical functional performance, population surveillance, walking

## Abstract

Gait speed is a simple, effective indicator of age-related disease and disability. We sought to examine the prevalence and trends of slow gait speed in older Americans. Our unweighted analytic sample included 12,427 adults aged ≥ 65 years from the 2006–2016 waves of the Health and Retirement Study. Gait speed was measured in participant residences. Persons with gait speed < 0.8 or <0.6 m/s were slow. Sample weights were used to generate nationally representative estimates. The overall estimated prevalence of slow gait speed with the <0.8 m/s cut-point was 48.6% (95% confidence interval (CI): 47.4–49.8) in the 2006–2008 waves yet was 45.7% (CI: 44.3–47.1) in the 2014–2016 waves, but this downward trend was not statistically significant (*p* = 0.06). The estimated prevalence of slowness with the <0.6 m/s cut-point was 21.3% (CI: 20.4–22.3) for the 2006–2008 waves, 18.5% (CI: 17.5–19.4) for the 2010–2012 waves, and 19.2% (CI: 18.2–20.2) for the 2014–2016 waves, but there were again no significant trends (*p* = 0.61). Our findings showed that the estimated prevalence of slow gait speed in older Americans is pronounced, and different cut-points largely inform how slowness is categorized. Continued surveillance of slowness over time will help guide screening for disablement and identify sub-populations at greatest risk for targeted interventions.

## 1. Introduction

By the year 2030, nearly 20% of the global population will be aged 60 years or older, and by the year 2050, the number of persons aged at least 80 years will triple to about 426 million [1]. In the United States, the older American population is likewise projected to increase by approximately 113% by 2030 [2]. With nearly a quarter of disease burden attributed to the older adult population, there will be a similar elevation in healthcare costs associated with age-related morbidity and disability [3,4]. Moreover, older adults experience physical dysfunction, which contributes to a loss of mobility and a decrease in quality of life [5]. Declining mobility may increase fall risk and limit basic self-care [6,7]. These characteristics contribute to the medical-related economic implications of aging due to the increased need for physician care, treatments, and hospital stays [8]. For example, it is estimated the average cost for fall-related injuries, which are related to mobility, is $30,000 with subsequent increases in costs as age elevates [9]. Accordingly, examining mobility tasks in clinical and translational research settings may help to serve as an indicator of future age-related disease and disability. Such ongoing surveillance of mobility may better inform healthcare providers by presenting insights for earlier referral to interventions that are germane to restoring mobility.

Gait speed is a wide-spread mobility assessment that evaluates the time to comfortably walk across a pre-specified distance. Gait speed is also feasible and relatively inclusive, such that little to no expenses are necessary to complete gait speed assessments, and persons are effectively asked to walk a short distance. Examinations of gait speed are an important clinical marker that is widely used to assess physical function and mobility during aging [10]. Gait speed outcomes compared to other functional measurements can be used as both a reliable and valid indicator of overall health implications that may need healthcare intervention [11]. Criterion-referenced standards categorize gait speed measures, and persons identified as categorically slow have an increased risk for several health conditions such as cognitive impairment, functional disability, and early all-cause mortality [12]. Likewise, the presence of cardiometabolic disease, neurocognitive impairment, and difficulty performing activities of daily living limitations may be present prior to the administration of gait speed assessments, thereby suggesting a bidirectional association. Given that slow gait speed represents poor physical performance and a more advanced stage of the disablement process, monitoring gait speed may provide unique health insights during aging.

Previous investigations have shown that the proportions of persons that have slow gait speed may differ based on their characteristics. For example, Manjavong et al. [13] suggest that approximately 76% of older adults in an outpatient tertiary hospital setting were considered slow, while others revealed that about 56% of older adults residing in urban areas might be slow [14]. Despite the importance of monitoring gait speed, and how gait speed changes over time, the prevalence of older adults in the United States who might be considered slow is not well understood. Surveillance of gait speed may serve as a valuable screening tool for age-related disease and disability [15]. Additionally, the assessment of gait speed compared to other physical performance assessments is time-efficient and cost-effective [16]. Examining the prevalence of slow gait speed at a population level may provide insights into the presence of mobility impairments in Americans, inform interventions for restoring mobility, and guide healthcare policy for the quickly growing older demographic. We sought to evaluate the prevalence and trends of slow gait speed in older Americans from 2006–2016.

## 2. Materials and Methods

### 2.1. Participants

We performed a secondary analysis of data from the 2006–2016 waves of the Health and Retirement Study (HRS) [17]. The HRS utilizes a longitudinal panel design to examine health and economic factors in Americans as they age [18]. New cohorts of participants enter the HRS and are followed until death. Utilizing the HRS as a data source for this study provides multidisciplinary data for a national sample of Americans, including a multistage probability design, geographical stratification, and oversampling for certain demographic groups [19]. Sampling weights are provided by the HRS for generating nationally representative data.

Core interviews in the HRS occur in waves every 2 years and response rates have regularly been >80% [20]. Starting in the 2006 wave, the nationally representative face-to-face interviews included physical measures such as gait speed. These detailed interviews were performed at alternating waves with a random half-sample of HRS participants, while the other half-sample completed core interviews [21]. Accordingly, we merged these waves of the HRS to ensure that the random half-samples completing gait speed testing were evaluated uniformly. More details about the HRS are available elsewhere [22].

### 2.2. Measures

Interviewers created a walking course in an open and preferably non-carpeted area in participant residences. A strip of tape was secured on the floor to signify the beginning and end points of the walking course after interviewers measured the distance. Using a standardized protocol, participants were advised to walk at their normal pace across the 2.5 m course. Participants then aligned their toes at the start line, and when interviewers prompted participants to start, they walked across the course. Interviewers stopped timing when the participant’s foot was touching the floor past the finish line. After the first trial was completed, the same procedures were executed for the second trial [23,24]. Those with a recent surgery, injury, or other health condition that may have influenced walking may not have engaged in gait speed testing. Persons with gait speed < 0.8 or <0.6 m/s were considered slow [25,26]. These cut-points effectively dichotomize persons with mobility limitations and those without such limitations [26]. More details about the walking speed assessment in the HRS are available elsewhere [27]. Participants also told interviewers their age, gender, race, and ethnicity.

### 2.3. Statistical Analysis

All analyses were performed with SAS 9.4 software (SAS Institute; Cary, NC, USA). HRS analytic guidelines informed our analyses, such that we used the survey weights which accounted for the complex sampling design to generate nationally representative estimates in the PROC SURVEYFREQ procedure [19]. The descriptive characteristics were presented as unweighted mean ± standard deviation for continuous variables and frequency (percentage) for categorical variables to improve interpretability.

Prevalence estimates for slow gait speed using both the <0.8 and <0.6 m/s cut-points were shown as a weighted percentage and 95% confidence interval (CI). These prevalence estimates for both cut-points were displayed as overall, and then stratified by age group (young-old: 65–74 years, middle-old: 75–84 years, old-old: ≥85 years), gender (male, female), race and ethnicity (Hispanic, non-Hispanic Black, non-Hispanic Other, non-Hispanic White) for each combined HRS wave (2006–2008, 2010–2012, 2014–2016). Additionally, we calculated a percent difference in the prevalence estimates for the stratified findings using the 2006–2008 waves as the reference. 

Individual multilevel logistic regression models analyzed trends in slow gait speed separately using the <0.8 m/s and <0.6 m/s cut-points with the survey weights included for overall slowness, age group, sex, race and ethnicity. Repeated measures of individual persons in multiple waves were modeled using a random intercept for each participant to account for the longitudinal design in the HRS. For each model, the binary response variable was slow gait speed. In the overall model, there was only a single explanatory variable for time (i.e., wave). An additional model adjusted for time, age group (reference: young-old), and the interaction between time and age group for evaluating trends by age group. Furthermore, a model adjusted for time, sex (reference: female), and a time-by-sex interaction quantified trends by sex. Another model included explanatory variables for time, race and ethnicity (reference: non-Hispanic White), and the interaction. An alpha level of 0.05 was utilized for all analyses.

## 3. Results

**Figure 1:**
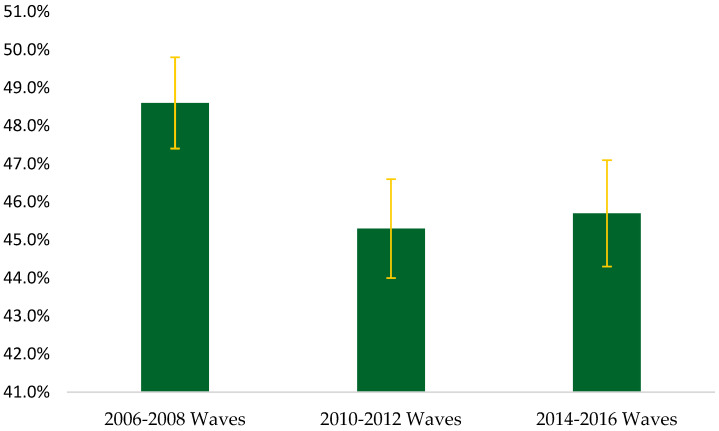
Overall estimated prevalence of slowness using a <0.8 m per second cut-point.

Table 1 shows the unweighted baseline descriptive characteristics of the 12,427 participants. Overall, persons were aged 72.7 ± 6.9 years and were mostly female (57.3%). Walking speed was 3.3 ± 1.7 s for young-old, 4.0 ± 2.7 s for middle-old, 5.0 s for old-old, 4.1 ± 3.3 s for Hispanic, 4.3 ± 2.5 s for non-Hispanic Black, 3.6 ± 2.6 s for non-Hispanic Other, 3.4 ± 1.8 s for non-Hispanic White, 3.8 ± 2.4 s for females, 3.3 ± 1.8 s for males. Figure 1 presents the overall prevalence of slow gait speed using a <0.8 m/s cut-point. The estimated prevalence of slow gait speed was 48.6% (CI: 47.4, 49.8), 45.3% (CI: 44.0, 46.6), and 45.7% (CI: 44.3, 47.1) in the 2006–2008, 2010–2012, and 2014–2016 waves, respectively. However, no significant trends in gait speed were observed over time (*p* = 0.06).

The estimated prevalence of slow gait speed by demographic characteristics using the <0.8 m/s cut-point is displayed in Table 2. The highest estimated prevalence of slow gait speed was 36.5% (CI: 35.0, 38.1) for the young-old group, 59.1% (CI: 57.1, 61.1) for the middle-old group in the 2006–2008 waves, and 79.1% (CI: 76.3, 81.9) for the old-old group in the 2014–2016 waves. Compared to those in the young-old age group, persons in the middle-old (*p* < 0.001) and old-old groups (*p* < 0.001) had a higher prevalence of slow gait speed. When examining the prevalence estimates by race and ethnicity using the <0.8 m/s cut-point, the estimated prevalence of slow gait speed in the most recent waves (2014–2016) was 64.2% (CI: 59.4, 69.0) for Hispanic, 73.1% (CI: 69.5, 76.6) for non-Hispanic Black, 46.4% (CI: 37.6, 55.1) for non-Hispanic Other, and 41.7% (CI: 40.2, 43.2) for non-Hispanic White. Compared to non-Hispanic White, Hispanic (*p* < 0.001) and non-Hispanic Black (*p* < 0.001) had a higher prevalence of slow gait speed. The highest estimated prevalence of slow gait speed using the <0.8 m/s cut-point was 54.0% (CI: 52.4, 55.6) for females in the 2006–2008 waves and 41.3% (CI: 39.5, 43.1) for males in the 2006–2008 waves. Males had a lower slow gait speed prevalence relative to females (*p* < 0.001). Table 3 presents the results for the slow gait speed trends analyses with the <0.8 m/s cut-point.

**Figure 2:**
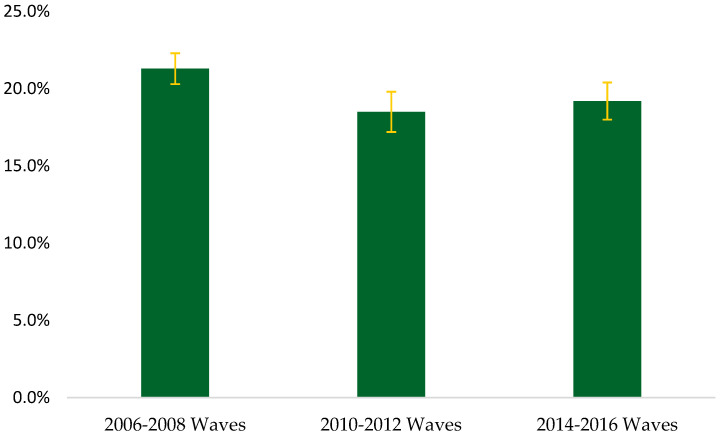
Overall estimated prevalence of slowness using a <0.6 m per second cut-point.

Figure 2 displays the overall estimated prevalence of slowness using a <0.6 m/s cut-point. The estimated prevalence of slow gait speed was 21.3% (CI: 20.4, 22.3) for the 2006–2008 waves, 18.5% (CI: 17.5, 19.4) for the 2010–2012 waves, and 19.2% (CI: 18.2, 20.2) for the 2014–2016 waves, but no significant trends were observed (*p* = 0.61). Table 4 presents the estimated prevalence of slow gait speed by demographic characteristics using a <0.6 m/s cut-point. Similar to the <0.8 m/s cut-point, the highest estimated prevalence of slowness with the <0.6 m/s was 13.5% (CI: 12.4, 14.6) for the young-old group, 26.7% (CI: 24.9, 28.5) for the middle-old group in the 2006–2008 waves, and 50.3% (CI: 46.8, 53.9) for the old-old group in the 2014–2016 waves. Compared to those in the young-old group, persons in the middle-old (*p* < 0.001) and old-old groups (*p* < 0.001) had a higher prevalence of slow gait speed. When evaluating the prevalence estimates by race and ethnicity using the <0.6 m/s cut-point, the estimated prevalence of slow gait speed in the 2014–2016 waves (most recent) was 30.4% (CI: 25.9, 34.9) for Hispanic, 37.8% (CI: 33.8, 41.7) for non-Hispanic Black, 22.2% (CI: 15.0, 29.4) for non-Hispanic Other, and 16.5% (CI: 15.5, 17.6) for non-Hispanic White. Relative to non-Hispanic White, Hispanic (*p* < 0.001) and non-Hispanic Black (*p* < 0.001) had a higher prevalence of slow gait speed. The highest estimated prevalence of slow gait speed was 25.5% (CI: 24.1, 26.9) for females and 15.7% (CI: 14.4, 17.0) for males, both in the 2006–2008 waves with the <0.6 m/s cut-point. Males had a lower gait speed prevalence compared to females (*p* < 0.001). The results for the slow gait speed trends analyses with the <0.6 m/s cut-point are shown in Table 5.

## 4. Discussion

Our results indicate that the estimated prevalence of slow gait speed in American adults aged at least 65 years nearly peaked at 49% when using the <0.8 m/s cut-point and was over 20% when utilizing the <0.6 m/s threshold from the 2006–2016 HRS population waves. The estimated prevalence of older adults with slow gait speed overall had no significant trend, and the estimated prevalence of slow gait speed was greatest in old-old adults and females, regardless of cut-point used. Moreover, persons identifying as Hispanic and non-Hispanic Black had an especially high estimated prevalence of slow gait speed. Our findings provide insights into the presence of slow gait speed among older adults in the United States, and how slow gait speed might be present in specific populations. The assessment of slow gait speed presents opportunities for improvement but remains a simple and inexpensive screening method for functional disability during aging.

The overall prevalence of slow gait speed in older adults has decreased from the 2006–2008 waves to the 2014–2016 waves, although these findings were not statistically significant. Not controlling for age-related morbidities and disabilities may have informed our prevalence estimates, and additional waves of HRS data may have yielded greater insights in this regard. Impairments in mobility tend to increase with age, and thus, it was not surprising that our findings indicated an elevation in gait speed prevalence with the advancing age group. For example, the older adult population aged 85 years and older generally displayed a greater prevalence of slow gait speed compared to young-old and middle-old adults. These findings can be attributed to physiological age-related declines such as frailty and cognitive impairment, and serve as a potential precursor to morbidities and mortality [28].

Gender may inform differences in gait speed, with women having a greater prevalence in slowness compared to men. This finding could be due to higher rates of osteoporosis in women than in men, wherein mobility and balance are potentially reduced, and fear of falling may be greater due to bone and joint frailty [29,30]. Furthermore, sarcopenia linked to menopausal hormonal deficiencies in estrogen presents adverse impacts related to musculoskeletal function in daily activities such as walking [31]. A higher proportion of women with obesity compared to men can further reduce mobility [32], and height differences between females and males is important to note for how stature may have influenced walking speed [33,34]. These findings pose health implications, suggesting women are a target group for the prevention and treatment of slow gait speed. Exercise and physical activity has shown promise to be an effective treatment in slowing the process of muscle loss, and pharmaceutical therapies such as the use of myostatin inhibitors can be utilized as an additional form of treatment [35].

Race and ethnicity were also factors, as there were high prevalence estimates for slow gait speed prevalence for Hispanic and non-Hispanic Black. These differences may be attributable to health disparities and the presence of other morbid conditions such as obesity, which in turn, may lead to dynapenia, joint pain, and instability during aging [36,37,38]. Furthermore, race and ethnicity factors and socioeconomic status can affect access to quality healthcare and treatment, thereby leading to an accelerated rate of aging [39]. Such implications for slow gait speed can be used as a predictive healthcare method for potential cognitive impairment, functional disability, and premature mortality, which can help inform the need for interventions [40]. Overall, interventions largely consist of resistance, aerobic, flexibility, and ambulation training in addition to task-oriented motor learning exercises. Moreover, community-based interventions could be useful for focusing on self-efficacy and care [41,42]. Due to its cost- and time-effectivity, the administration of gait speed as a preventative and diagnostic tool in healthcare settings is particularly useful.

Some limitations should be noted. Standardization in residence settings of participants was not controlled for such as carpet or hard flooring where gait speed was performed. Additionally, the footwear of participants was not similar, which has the capacity to induce biomechanical impacts on gait [43]. The HRS utilized a 2.5 m distance for ascertaining walking speed, but other distances and protocols may have influenced the findings [44]. Because these gait speed data were collected from 2006–2016, there is a possibility that select participants did not subsequently continue gait speed measurements due to morbidities, movement in care facilities, and death, which may have implications on our findings. Despite these limitations, our investigation revealed slow gait speed estimates amongst older Americans and vulnerable populations who are a target for prevention and intervention. We recommend gait speed continues to be monitored alongside other cognitive and physical (e.g., handgrip strength) indicators as the older adult population increases to help inform the need for intervention.

## 5. Conclusions

The overall estimated prevalence of slow gait speed in Americans aged at least 65 years peaked at approximately 49% when using the <0.8 m/s cut-point and was >20% when categorizing slowness with the <0.6 m/s cut-point for the 2006–2016 HRS populations waves. The prevalence estimates of older adults who displayed slowness generally increased from the young-old age category to the old-old age category. Females and non-Hispanic Black had higher slow gait speed prevalence estimates, which present target populations for potential healthcare intervention. Examining gait speed remains a simple screening method for age-related disease and disability, and using different cut-points will inform how persons are categorized as slow. As the older American population continues increasing, health conditions that are germane to this age demographic will likewise elevate. Therefore, gait speed assessments could be a valuable screening tool for guiding healthcare providers and relevant policies in helping older Americans live longer with independence.

## Figures and Tables

**Table 1:** Unweighted descriptive characteristics of the participants.

	Overall (n = 12,427)
Age (years)	72.7 ± 6.9
Age Category (n (%))	
Young-Old (n (%))	8214 (66.1)
Middle-Old (n (%))	3258 (26.2)
Old-Old (n (%))	955 (7.7)
Sex (n (%))	
Male (n (%))	5301 (42.7)
Female (n (%))	7126 (57.3)
Race and Ethnicity (n (%))	
Hispanic	1120 (9.0)
Non-Hispanic Black	1648 (13.3)
Non-Hispanic Other	270 (2.1)
Non-Hispanic White	9389 (75.6)
Health Conditions	2.3 ± 1.4
Walk Speed (seconds)	3.6 ± 2.1

Note: Health conditions included a count of self-reported healthcare-provider-diagnosed hypertension, diabetes, cancer, lung disease, heart disease, stroke, psychiatric problems, and arthritis (n = 12,426).

**Table 2:** Estimated prevalence of slowness by demographic characteristics using a <0.8 m per second cut-point.

Variable	Weighted Frequency	Weighted Prevalence (%)	95% Confidence Interval	∆% ^†^
Age Group				
Young-Old				
2006–2008 Waves	5,403,022	36.5	35.0, 38.1	-
2010–2012 Waves	5,880,135	34.5	32.7, 36.2	−2.0
2014–2016 Waves	7,384,681	35.1	33.2, 37.0	−1.4
Middle-Old				
2006–2008 Waves	5,539,249	59.1	57.1, 61.1	-
2010–2012 Waves	5,398,282	53.7	51.7, 55.6	−5.4
2014–2016 Waves	5,707,398	54.9	52.9, 56.9	−4.2
Old-Old				
2006–2008 Waves	2,221,729	76.1	72.8, 79.4	-
2010–2012 Waves	2,519,977	74.8	71.6, 77.9	−1.3
2014–2016 Waves	3,047,428	79.1	76.3, 81.9	3.0
Race & Ethnicity				
Hispanic				
2006–2008 Waves	1,061,208	63.6	59.2, 68.1	-
2010–2012 Waves	1,028,953	57.7	52.3, 63.1	−5.9
2014–2016 Waves	1,560,875	64.2	59.4, 69.0	0.6
Non-Hispanic Black				
2006–2008 Waves	1,304,420	69.4	65.9, 72.8	-
2010–2012 Waves	1,654,638	74.5	71.2, 77.8	5.1
2014–2016 Waves	1,890,268	73.1	69.5, 76.6	3.7
Non-Hispanic Other				
2006–2008 Waves	255,375	51.7	41.9, 61.5	-
2010–2012 Waves	296,928	45.5	36.3, 54.7	−6.2
2014–2016 Waves	437,165	46.4	37.6, 55.1	−5.3
Non-Hispanic White				
2006–2008 Waves	10,542,997	45.8	44.5, 47.1	-
2010–2012 Waves	10,817,875	41.9	40.5, 43.3	−3.9
2014–2016 Waves	12,251,199	41.7	40.2, 43.2	−4.1
Gender				
Females				
2006–2008 Waves	8,402,444	54.0	52.4, 55.6	-
2010–2012 Waves	8,732,664	51.2	49.5, 52.9	−2.8
2014–2016 Waves	10,002,946	51.2	49.4, 53.0	−2.8
Males				
2006–2008 Waves	4,761,556	41.3	39.5, 43.1	-
2010–2012 Waves	5,065,730	37.7	35.8, 39.7	−3.6
2014–2016 Waves	6,136,561	38.9	36.9, 41.0	−2.4

^†^ 2006–2008 waves were the reference.

**Table 3:** Results for the slow gait speed trends analyses with the <0.8 m per second cut-point.

	Estimate	95% Confidence Interval	*p*-Value
**Overall Model**			
Intercept	−0.40	−0.80, −0.01	0.04
Wave	0.18	−0.01, 0.36	0.06
**Model 1**			
Intercept	−0.45	−0.54, −0.35	<0.001
Wave	0.01	−0.04, 0.06	0.65
Middle-Old	0.77	0.62, 0.92	<0.001
Old-Old	1.66	1.42, 1.89	<0.001
Wave*Middle-Old	−0.03	−0.10, 0.04	0.44
Wave*Old-Old	−0.02	−0.12, 0.09	0.71
**Model 2**			
Intercept	0.87	0.36, 1.39	<0.001
Wave	0.13	−0.11, 0.37	0.28
Male	−2.90	−3.70, −2.20	<0.001
Wave*Male	0.08	−0.29, 0.45	0.69
**Model 3**			
Intercept	−1.20	−1.60, −0.76	<0.001
Wave	0.03	−0.18, 0.24	0.79
Hispanic	4.23	2.77, 5.69	<0.001
Non-Hispanic Black	5.36	4.13, 6.60	<0.001
Non-Hispanic Other	0.78	−2.20, 3.71	0.60
Wave*Hispanic	−0.02	−0.68, 0.64	0.94
Wave*Non-Hispanic Black	0.40	−0.17, 0.97	0.16
Wave*Non-Hispanic Other	0.08	−1.20, 1.38	0.90

**Table 4:** Prevalence estimates of slow gait speed by demographic characteristics using a <0.6 m per second cut-point.

Variable	Weighted Frequency	Weighted Prevalence (%)	95% Confidence Interval	∆% ^†^
Age Group				
Young-Old				
2006–2008 Waves	2,000,914	13.5	12.4, 14.6	
2010–2012 Waves	1,848,315	10.8	9.7, 11.9	−2.7
2014–2016 Waves	2,342,180	11.1	9.9, 12.3	−2.4
Middle-Old				
2006–2008 Waves	2,500,642	26.7	24.9, 28.5	
2010–2012 Waves	2,312,662	23.0	21.3, 24.6	−3.7
2014–2016 Waves	2,500,728	24.0	22.3, 25.7	−2.7
Old-Old				
2006–2008 Waves	1,280,654	43.9	40.1, 47.6	
2010–2012 Waves	1,478,506	43.8	40.1, 47.5	−0.1
2014–2016 Waves	1,940,661	50.3	46.8, 53.9	6.4
Race & Ethnicity				
Hispanic				
2006–2008 Waves	569,762	34.1	30.0, 38.3	
2010–2012 Waves	503,120	28.2	23.3, 33.1	−5.9
2014–2016 Waves	739,686	30.4	25.9, 34.9	−3.7
Non-Hispanic Black				
2006–2008 Waves	725,509	38.6	34.9, 42.2	-
2010–2012 Waves	829,684	37.3	33.6, 41.0	−1.3
2014–2016 Waves	978,070	37.8	33.8, 41.7	−0.8
Non-Hispanic Other				
2006–2008 Waves	112,414	22.7	13.9, 31.5	-
2010–2012 Waves	142,702	21.8	13.8, 29.9	−0.9
2014–2016 Waves	209,409	22.2	15.0, 29.4	−0.5
Non-Hispanic White				
2006–2008 Waves	4,374,525	19.0	17.9, 20.0	-
2010–2012 Waves	4,163,977	16.1	15.1, 17.1	−2.9
2014–2016 Waves	4,856,404	16.5	15.5, 17.6	−2.5
Gender				
Females				
2006–2008 Waves	3,969,203	25.5	24.1, 26.9	-
2010–2012 Waves	3,839,996	22.5	21.1, 23.9	−3.0
2014–2016 Waves	4,446,626	22.7	21.3, 24.2	−2.8
Males				
2006–2008 Waves	1,813,007	15.7	14.4, 17.0	-
2010–2012 Waves	1,799,487	13.4	12.1, 14.7	−2.3
2014–2016 Waves	2,336,943	14.8	13.4, 16.2	−0.9

^†^ 2006–2008 waves were the reference.

**Table 5:** Results for the slow gait speed trends analyses with the <0.6 m per second cut-point.

	Estimate	95% Confidence Interval	*p*-Value
**Overall Model**			
Intercept	−6.6	−7.0, −6.3	<0.001
Wave	0.04	−0.12, 0.20	0.61
**Model 1**			
Intercept	−1.7	−1.9, −1.6	<0.001
Wave	−0.03	−0.09, 0.02	0.25
Middle-Old	0.69	0.50, 0.88	<0.001
Old-Old	1.54	1.24, 1.83	<0.001
Wave*Middle-Old	0.02	−0.07, 0.11	0.63
Wave*Old-Old	0.06	−0.07, 0.20	0.37
**Model 2**			
Intercept	−5.6	−6.0, −5.1	<0.001
Wave	−0.05	−0.25, 0.16	0.66
Male	−2.6	−3.3, −1.9	<0.001
Wave*Male	0.19	−0.14, 0.52	0.25
**Model 3**			
Intercept	−7.5	−7.8, −7.1	<0.001
Wave	−0.03	−0.21, 0.16	0.77
Hispanic	4.00	2.75, 5.26	<0.001
Non-Hispanic Black	5.19	4.14, 6.23	<0.001
Non-Hispanic Other	0.45	−2.10, 3.04	0.73
Wave*Hispanic	−0.19	−0.75, 0.38	0.51
Wave*Non-Hispanic Black	−0.09	−0.57, 0.39	0.72
Wave*Non-Hispanic Other	0.36	−0.79, 1.51	0.53

## Data Availability

Data utilized in this investigation are publicly available from the Health and Retirement Study website.
